# Safety of falciparum malaria diagnostic strategy based on rapid diagnostic tests in returning travellers and migrants: a retrospective study

**DOI:** 10.1186/1475-2875-11-377

**Published:** 2012-11-16

**Authors:** Isabelle Anne Rossi, Valérie D’Acremont, Guy Prod’Hom, Blaise Genton

**Affiliations:** 1 Travel Clinic, Department of Ambulatory Care and Community Medicine, University of Lausanne, Lausanne, CH-1011, Switzerland; 2 Swiss Tropical and Public Health Institute, Basel, CH-4002, Switzerland; 3 Global Malaria Programme, World Health Organization, Geneva, CH-1211, Switzerland; 4 Institute of Microbiology, University of Lausanne, Lausanne, CH-1011, Switzerland; 5 Infectious Disease Service, Department of Internal Medicine, University Hospital, Lausanne, CH-1011, Switzerland

**Keywords:** Malaria, Rapid diagnostic tests, Diagnosis, Travellers, Migrants

## Abstract

**Background:**

Rapid diagnostic tests for malaria (RDTs) allow accurate diagnosis and prompt treatment. Validation of their usefulness in travellers with fever was needed. The safety of a strategy to diagnose falciparum malaria based on RDT followed by immediate or delayed microscopy reading at first attendance was evaluated in one referral hospital in Switzerland.

**Methods:**

A retrospective study was conducted in the outpatient clinic and emergency ward of University Hospital, covering a period of eight years (1999–2007). The study was conducted in the outpatient clinic and emergency ward of University Hospital. All adults suspected of malaria with a diagnostic test performed were included. RDT and microscopy as immediate tests were performed during working hours, and RDT as immediate test and delayed microscopy reading out of laboratory working hours. The main outcome measure was occurrence of specific complications in RDT negative and RDT positive adults.

**Results:**

2,139 patients were recruited. 1987 had both initial RDT and blood smear (BS) result negative. Among those, 2/1987 (0.1%) developed uncomplicated malaria with both RDT and BS positive on day 1 and day 6 respectively. Among the 152 patients initially malaria positive, 137 had both RDT and BS positive, four only BS positive and five only RDT positive (PCR confirmed) (six had only one test performed). None of the four initially RDT negative/BS positive and none of the five initially BS negative/RDT positive developed severe malaria while 6/137 of both RDT and BS positive did so. The use of RDT allowed a reduction of a median of 2.1 hours to get a first malaria test result.

**Conclusions:**

A malaria diagnostic strategy based on RDTs and a delayed BS is safe in non-immune populations, and shortens the time to first malaria test result.

## Background

In many industrialized countries, imported malaria accounts for a significant burden of disease and mortality every year [1-3]. Prompt treatment of malaria is essential because of its potentially rapid and fatal course. Quick documentation of *Plasmodium* parasitaemia in the blood is the mainstay to initiate appropriate treatment. However, accurate diagnosis remains a challenge. Diagnosis by microscopy is still considered as the gold standard, but turnaround time often exceeds 2–3 hours and accuracy is acceptable only when performed by experienced microscopists [4,5]. Rapid diagnostic tests for malaria (RDTs) have recently emerged as an alternative to microscopy [6]. Their accuracy in endemic and non-endemic countries has been extensively evaluated and confirmed in three meta-analyses [7-9]. Furthermore, their performance, even in the industrialized world, is often superior to blood smears (BS) performed under routine clinical laboratory conditions [10].

Based on the available evidence of excellent diagnostic performance of RDTs, insufficient sensitivity of routine microscopy, especially in non immune travellers [11], and the identification of reliable clinical and laboratory predictors of malaria [12], a new strategy for the diagnosis of malaria in febrile patients returning from endemic countries was introduced in October 1999. The strategy was based on RDT as immediate test and delayed microscopy in patients without danger signs attending out of hours.

In this report, an evaluation of the safety (incidence of complications or death) of a malaria diagnostic strategy based on RDTs and implemented under routine conditions for returning travellers and migrants in a non endemic country, over an eight-year period is provided. The safety of using RDTs as main diagnostic tool has already been demonstrated in many patients of semi-immune populations in endemic countries [13-15].

## Methods

### Subjects and study setting

Files of all adult patients (≥ 16 years) attending the outpatient clinic and emergency ward of the University Hospital in Lausanne between October 1999 and August 2007 who underwent a malaria test were reviewed. Children were not included since they are managed in a different location. Only patients who had a positive RDT for falciparum only or mixte infection were included in the study. Indeed, severe malaria due to non-falciparum mono-infection is rare and, at the time of the study, the sensitivity for *Plasmodium vivax* of available RDT brands was rather low.

### Strategy assessed

**Figure 1:**
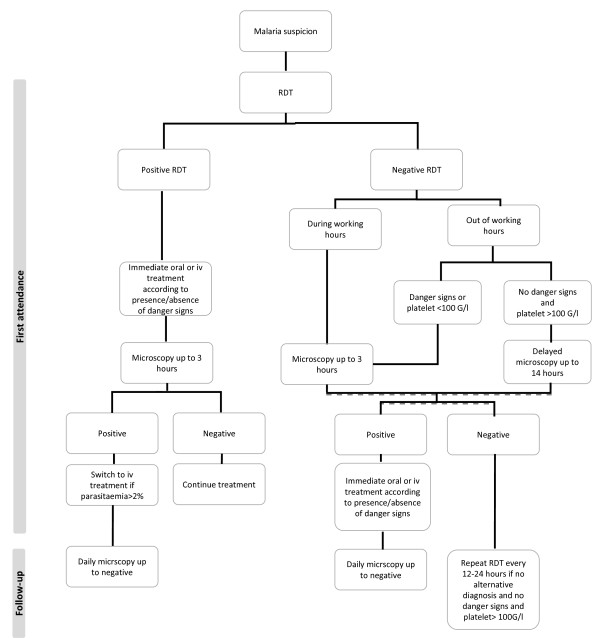
Malaria diagnostic strategy during and out of working hours.

The diagnostic strategy included different procedures determined by the opening hours of the parasitology laboratory of the University Hospital. All patients returning from a malaria endemic country and complaining of fever were to undergo first an RDT. If they presented during working hours, the microscopical examination was to be performed immediately or at least within three hours. Out of hours and during weekends, patients were to undergo just an RDT if there was no danger sign. If the RDT was negative, the microscopy reading was delayed until the next day (up to 14 hours). If there was a danger sign at first attendance, patients were to undergo an RDT followed by a microscopical examination within three hours, irrespective of parasitology laboratory opening hours. The presence of thrombocytopaenia <100 G/l was also considered sufficient to warrant an immediate BS, since it has proved to be a strong diagnostic predictor of malaria (positive likelihood ratio of 11) [12,16]. When the RDT was positive, the patient was to be given immediately appropriate anti-malarial treatment (oral or intravenous) according to the presence/absence of severe malaria and the microscopical examination was to follow immediately (see detailed strategy in Figure 1). The standard treatment for uncomplicated malaria was mefloquine (Lariam®, Roche; Mephaquine®, Mepha) from 1999–2002 and the combination of artemether/lumefantrine (Riamet®, Novartis) from 2003 onwards [17]. For severe malaria the standard treatment was quinine plus doxycycline intravenously all along the study. The treatment was adjusted following reception of the microscopy result (switch to intravenous treatment when parasite density was >2%). RDTs used were ICT Malaria Pf/Pv (ICT-Amrad, Sydney, Australia) until January 2006 and then OptiMAL® (Diamed, Cressier, Switzerland). Regarding microscopy, only thin smears were available at the time of the study, which were declared negative after reading during 20 minutes.

### Definitions

A case of *Plasmodium falciparum* malaria was defined as a patient with at least one of the tests (RDT or microscopical examination) positive for *P. falciparum* only or mixed infection, irrespective of any other incidental diagnosis. Patients with danger sign(s) were defined as patients having at least one of the following signs: poor general condition, any neurological sign, respiratory distress, hypotension (systolic PA < 90 mmHg), shock (systolic PA < 70 mmHg), severe renal insufficiency (creatinine >250 μmol/l), severe hepatic insufficiency (INR ≥ 1.5 and any degree of mental alteration), severe anaemia (< 80 g/dl), bleeding sign, hypoglycaemia (<3.9 mmol/l). These criteria, plus a few others (repeated vomiting, high parasite density (>2%), failure with previous anti-malarial treatment, poor compliance, alone at home, pregnancy) are also used for deciding about admission of the patient. Patient who do not fulfill these criteria are managed on an ambulatory base [18]. Complications were defined as the new onset of danger signs or progression to severe malaria (according to the WHO case definition malaria), [19] that was not present at enrollment and occurred during follow-up.

### Procedures for retrospective analysis

Demographic information and details on time of availability of RDT test result and microscopy result were extracted from patient’s file and laboratory database. The time between RDT and microscopy result availability could then be calculated. Because the guidelines in the University Hospital in Lausanne specify that the anti-malarial treatment should be initiated as soon as the first malaria test is positive, the latter corresponds closely to the time to onset of appropriate treatment saved when using RDT rather than microscopy alone. To assess safety, the occurrence of death (any cause) during follow-up in both malaria and non-malaria patients as well as the occurrence of severe malaria (according to WHO case definition) [19] in malaria patients was calculated. The number of initially negative patients (by RDT and microscopy) who turned positive during follow-up (so called ‘missed malaria’), and the consequence on outcome were also recorded.

Detailed clinical information was extracted from the file for all patients who were once malaria positive to ascertain severe case definition. Follow-up was done up to the last consultation (negative BS and recovery or recovery only for those who were RDT positive but always microscopy negative). Such detailed clinical assessment was not done for patients always malaria negative since this was not related to study objectives. To ascertain that none of these latter patients died of malaria outside the University hospital (at home or elsewhere), all death records of the Federal Office of Statistics that mentioned malaria as a cause for the same period were reviewed. These data were cross-checked with malaria death records of the Federal and Cantonal Office of Public Health.

To assess the impact on the safety (occurrence of severe malaria) of full compliance or not to the diagnostic strategy, health outcomes according to the tests done (RDT or microscopy) and timelines (within 3 or 14 hours) depending on arrival (working hours or out of hours) for the malaria positive patients were analysed.

## Results

Between October 1999 and August 2007 a total of 2,190 patients older than 16 years were tested for malaria (as per laboratory records). 41 were excluded because they were positive for a non-falciparum species only. 10 patients positive for *P. falciparum* were also excluded, six because clinical files could not be retrieved and four because the pre-defined diagnostic strategy could not be used. For two of them, diagnosis was indeed fortuitous during examination of a blood smear; two other cases had already taken a full treatment (one had only gametocytes, the other a positive RDT). None of these four excluded cases developed severe malaria. 2,139 patients were thus included in the present retrospective analysis.

The demographic characteristics of the 154 falciparum malaria patients were as follows: 60% were men; mean age was 38.9 years; 79% were returning travellers, 9% migrants and 12% expatriates; 97% contracted malaria in sub-Saharan Africa. Among the 126 travellers who should have taken malaria chemoprophylaxis according to the recommendations, only three had regularly taken the recommended chemoprophylactic agent. Clinically, the mean duration of symptoms before consultation was 3.9 days; 31% had a maximal parasite density of <0.1%, 36% of 0.1-0.9%, 24% of 1.0-4.9 and 8% ≥5%; 25% had at least one co-morbidity; 30% had at least one danger sign and 17.5% had severe malaria at first attendance.

### Malaria test results at first attendance

**Figure 2:**
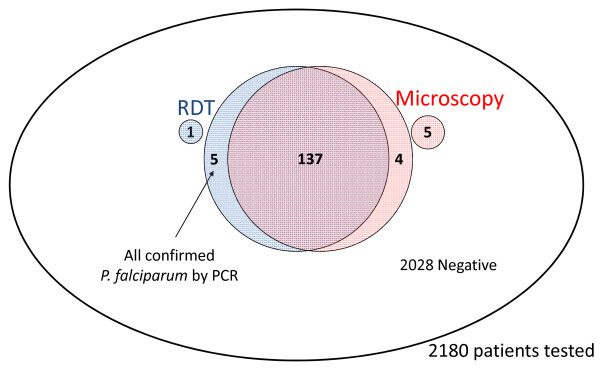
Malaria test results at first attendance: concordance between RDT and microscopy results.

Initial malaria tests results are pictured in Figure 2. 1,987 (92.9%) had both RDT and BS result negative. 152 (7.6%) were malaria positive: 137 by RDT and microscopy, four by microscopy only and five by RDT only (one was tested by RDT only and five by microscopy only). Full agreement between the two methods was thus 94%. The four patients who were negative by RDT but positive by microscopy had a parasite density of 0.4% for one and <0.1% for the other three. The five patients who were positive by RDT but negative by microscopy were all confirmed positive by PCR performed at a later stage [20].

### Malaria occurrence during follow-up and health outcomes according to initial test result

**Figure 3:**
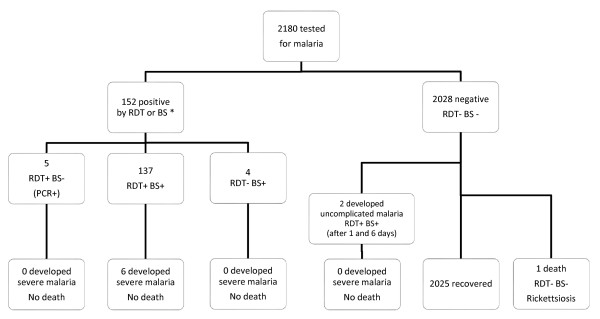
**Malaria occurrence during follow-up and health outcomes according to initial test results.** One was tested by RDT only and five by microscopy only; the six recovered unventfully (not presented in the Figures).

Malaria occurrence during follow-up of initially negative patients and number of severe malaria and deaths according to initial malaria test results are detailed in Figure 3.

Among the 1,987 initially negative patients, two (0.1%) developed uncomplicated malaria with both RDT and BS positive on day 1 and day 6 respectively; they both recovered uneventfully. One out of 1,987 negative patient died; both RDT and BS results were always negative; the cause of death was rickettsiosis (case report published elsewhere) [21]. Among the 152 patients malaria positive at first attendance, no death occurred. None of the four initially RDT negative /BS positive (one had danger signs upon admission) and five initially RDT positive /BS negative (one had danger signs upon admission) developed severe malaria during follow-up while 6/137 of both RDT and BS positive did so (43/137 had already danger signs upon admission). Of note, one patient with RDT positive but negative BS result had severe malaria at the time of diagnosis (three days after admission). He was not suspected of malaria on the day of admission, even if he was coming back from expatriation in Democratic Republic of Congo, since he was hospitalized for a surgical procedure (exploratory laparotomy). Three days after admission and surgical procedure, he developed fever and impairment of consciousness. An RDT at that moment was done and found positive. Treatment with intravenous quinine was started but wrongly interrupted the day after, due to the fact that clinicians did not trust the RDT positive result in the presence of a negative BS. Fifteen days later, the patient developed again signs of severe malaria and the BS showed a parasitaemia of 4.1%. Recovery was uneventful after appropriate treatment.

### Time between availability of RDT result and microscopy result

Information to calculate the time between RDT result and microscopy result was available for 125 (85.6%) malaria positive cases. The median time to get the BS result after having had the RDT result was 2.1 hours (IQ 1.5-3.6 hours, range 32 minutes-72.8 hours) [2.0 hours (IQ 1.6-2.6 hours, range 32 minutes-72.8 hours) during laboratory working hours and 2.1 hours (IQ 1.4-5.1 hours, range 33 minutes-70.8 hours) out of hours]. Of note the median time between the blood sample and RDT result was 15 minutes (IQ 15–50 minutes, range 15 minutes-5.8 hours).

### Compliance to the timelines defined by the diagnostic strategy

Overall non-compliance to timelines defined in the diagnostic strategy was 31.5% (40/127). It was 20% (10/50) for patients attending during working hours and 39% (30/77) for those attending out of hours (p = 0.04). Non-compliance to timelines was 29% (20/69) for patients with danger signs and 34.5% (20/58) for those without danger signs (p = 0.64). Non-compliance to timeline recommendations (for those with or without danger signs) did not affect heath outcome.

### Relation of timelines to occurrence of severe malaria

Median time before getting a BS result was 2.1 hours (IQ 1.4-3.5, range 32 minutes-72.8 hours) for the cases without any complication and 3.2 hours (IQ 2.1-16.7, range 1.6-27.8) for the six cases who developed severe malaria after admission (p = 0.14). Among the four patients with negative RDT but positive microscopy, the delay between the RDT and the BS result was 14, 18.3, 37.7 and 70.8 hours. One of the four had a danger signs at first attendance and none developed severe malaria (as mentioned above).

Among the six cases that developed severe malaria after admission, all six had a positive RDT and BS at first testing. Three had microscopy reading within three hours after RDT result; for the other three, the delay to get microscopy results was respectively of 4.3, 17 and 27 hours. One had no danger sign, three had at least one danger sign at first attendance and another two had thrombocytopaenia <100 G/L. Among those with danger signs or thrombocytopaenia, all except one received intravenous quinine at first place. All six cases who developed severe malaria after admission recovered uneventfully.

## Discussion

The present study provides, for the first time, evidence that a malaria diagnostic strategy based on a RDT followed by immediate or delayed microscopy reading at first attendance is safe and does not expose travellers or migrants to an increased risk of severe malaria or death. These findings can probably be generalized to most setting in non-endemic countries since they derive from data collected in predominantly non-immune patient population under routine clinical and laboratory conditions of an ordinary outpatient clinical and emergency hospital ward. No patient with a negative RDT developed severe malaria, despite a planned delay before getting the blood smear results out of hours. This was true even when established timelines between RDT and microscopy were not complied to. The use of RDTs was not associated with the development of complications since all 6 cases who developed severe malaria after admission had a positive RDT at first testing. The availability nowadays of artemisinin-based combination therapy (ACT) for the treatment of uncomplicated malaria renders the strategy with RDT even safer since ACT is very effective and well-tolerated [17,22]. ACT should be given in the outpatient department or emergency ward immediately after a positive RDT result while waiting for the BS result, even in uncomplicated cases. Similarly, quinine should be administered straight after a positive RDT result for severe cases.

Even if the study was not designed to validate the accuracy and performance of RDTs, which has already been extensively demonstrated, RDTs were as good as microscopy to diagnose malaria. Indeed 5 malaria diagnoses were based on positive RDT results only (negative blood slide) and 4 on blood smear results only (negative RDT result). Recently, Gillet *et al.*[23,24] and Luchavez *et al.*[25] demonstrated that the prozone effect (false-negative or false-low results, due to an excess of either antigen or antibody) exists, but has so far only been described with histidine-rich protein 2 tests [23,24]. Negative results were rare compared to an increase in test line intensity after dilution. In our study, none of the negative RDT results was explained by the prozone effect. This strategy of performing an immediate blood smear in the presence of danger signs or a thrombocytes count < 100 G/l (higher pre-test probability) should prevent delay in the diagnosis of malaria in case of false negative RDT result in the presence of hyperparasitaemia.

Diagnostic strategies based on RDT have been adopted in other centres managing non-immune patients [10,26] but, this is the first study assessing the safety of such strategy. Because of the retrospective design of the study, we were able to assess the strategy under routine clinical and laboratory practice. As imported malaria is a rare disease and severe malaria even rarer, it was not possible to perform a non-inferiority trial comparing the strategy with and without RDT. Also there are concerns not to use RDTs in a setting where not all laboratory technicians are familiar with malaria parasites, especially out of hours, which might have resulted in missed malaria, and hence higher rate of complications.

The less rigorous follow-up of the malaria negative patients is a limitation in the overall assessment. However, it is highly unlikely that secondary malaria cases were missed after having attended the outpatient clinic since feverish patients are advised to come back daily to repeat malaria tests, especially so if symptoms persist or worsen. In addition, the University Hospital in Lausanne has a long tradition of reference centre for travel related diseases in the area and is easily accessible. At least, secondary malaria deaths that would have occurred outside the hospital have been virtually excluded by our investigation of malaria death records in the region. This study was undertaken in one hospital only and should be repeated in different settings to accumulate more evidence. Since non-falciparum malaria patients and children were excluded, the safety of this strategy should be confirmed for malaria due to other species (especially so since RDT of last generation do detect vivax with excellent sensitivity) and in a paediatric population [27].

In conclusion, this study - conducted in a routine clinical and laboratory non-endemic setting without 24-hour expert microscopy available – provides some evidence that a malaria diagnostic strategy based on RDTs followed by immediate or delayed microscopy reading is safe. Indeed no patients with a negative RDT developed severe malaria or died. This study adds information about the safety of a malaria diagnostic strategy based on RDTs, of which accuracy and performance have been extensively demonstrated. There was also a clear benefit of using RDT, as it allowed decreasing significantly the delay before getting a test result (and thus onset of appropriate treatment), even during laboratory working hours and increasing overall sensitivity when combined with microscopy. The results of this analysis provide evidence and lessons for considering large-scale implementation of malaria diagnostic strategies that include RDTs in non-endemic settings.

## Competing interests

Authors declare that they have no competing interests.

## Authors' contributions

IAR was responsible for the design of the retrospective investigation, data extraction, entry, management and analysis, interpretation of results and writing the manuscript. VDA contributed to the development and design of the strategy, clinical supervision, interpretation of the results and writing the manuscript. GP’H contributed to the development and design of the strategy, and data extraction. BG contributed to the development and design of the strategy, clinical supervision, interpretation of the results and writing the manuscript. All authors read and approved the final manuscript.
